# Effect of A-Site Cation Ordering on Chemical Stability, Oxygen Stoichiometry and Electrical Conductivity in Layered LaBaCo_2_O_5+δ_ Double Perovskite

**DOI:** 10.3390/ma9030154

**Published:** 2016-03-03

**Authors:** Carlos Bernuy-Lopez, Kristin Høydalsvik, Mari-Ann Einarsrud, Tor Grande

**Affiliations:** Department of Material Science and Engineering, NTNU Norwegian University of Science and Technology, Trondheim NO-7491, Norway; kristin.hoydalsvik@ntnu.no (K.H.); mari-ann.einarsrud@ntnu.no (M.-A.E.); tor.grande@ntnu.no (T.G.)

**Keywords:** layered double perovskite (LDP), cation and anion ordering, chemical expansion, solid oxide fuel cells

## Abstract

The effect of the A-site cation ordering on the chemical stability, oxygen stoichiometry and electrical conductivity in layered LaBaCo_2_O_5+δ_ double perovskite was studied as a function of temperature and partial pressure of oxygen. Tetragonal A-site cation ordered layered LaBaCo_2_O_5+δ_ double perovskite was obtained by annealing cubic A-site cation disordered La_0.5_Ba_0.5_CoO_3-δ_ perovskite at 1100 °C in N_2_. High temperature X-ray diffraction between room temperature (RT) and 800 °C revealed that LaBaCo_2_O_5+δ_ remains tetragonal during heating in oxidizing atmosphere, but goes through two phase transitions in N_2_ and between 450 °C and 675 °C from tetragonal *P4/mmm* to orthorhombic *Pmmm* and back to *P4/mmm* due to oxygen vacancy ordering followed by disordering of the oxygen vacancies. An anisotropic chemical and thermal expansion of LaBaCo_2_O_5+δ_ was demonstrated. La_0.5_Ba_0.5_CoO_3-δ_ remained cubic at the studied temperature irrespective of partial pressure of oxygen. LaBaCo_2_O_5+δ_ is metastable with respect to La_0.5_Ba_0.5_CoO_3-δ_ at oxidizing conditions inferred from the thermal evolution of the oxygen deficiency and oxidation state of Co in the two materials. The oxidation state of Co is higher in La_0.5_Ba_0.5_CoO_3-δ_ resulting in a higher electrical conductivity relative to LaBaCo_2_O_5+δ_. The conductivity in both materials was reduced with decreasing partial pressure of oxygen pointing to a p-type semiconducting behavior.

## 1. Introduction

Layered double perovskite (LDP) materials with mixed ionic-electronic conductivity and high catalytic activity have been heavily investigated because of their potential application in electrochemical devices such as solid oxide fuel cells (SOFC) [1,2,3,4,5,6,7,8] or gas separation membranes [9,10]. These materials are described with a general formula *Ln*Ba*M*_2_O_5+δ_ where *Ln* is a lanthanide metal or Y and *M* is a transition metal. In this double perovskite AA′B_2_O_6_-type crystal structure, *Ln* and Ba are occupying the A-site in the perovskite structure while *M* occupies the B-site. A-site cation ordering is adopted due to the large difference in size of Ba and *Ln* with *Ln*O and BaO layers in dodecahedral coordination separated by *M*O_6_ layers in octahedral coordination [11]. The cation ordering leads to a tetragonal symmetry *P4/mmm* (a_p_ × a_p_ × 2 a_p_) in comparison with the cation disordered *Ln*_0.5_Ba_0.5_*M*O_3-δ_ counterpart which adopts a cubic symmetry [12,13]. A-site cation ordering has been shown to be beneficial for the performance of the material as a SOFC cathode. For example, A-site cation ordered GdBaMnFeO_5+δ_ has been reported to possess superior cathode SOFC properties compared with the cubic perovskite Gd_0.5_Ba_0.5_Mn_0.5_Fe_0.5_O_3-δ_ counterpart [14].

In addition to cation ordering, oxygen deficiency at elevated temperatures may also lead to ordering of oxygen vacancies, e.g., for LaBaCo_2_O_5+δ_ [15], PrBaCo_2_O_5+δ_ [16] or GdBaCo_2_O_5+δ_ an orthorhombic structure with space group *Pmmm* (*a_p_* × 2 *a_p_* × 2 *a_p_*) is adopted when the oxygen vacancies reaches a certain level dependent on the type of lanthanide and transition metals [13]. The oxygen vacancies are located in the *Ln*O layer of the double perovskite structure in order to compensate for the size difference between *Ln* and Ba [5,15,17,18,19]. In contrast to A-site cation ordering, the oxygen vacancy ordering is not beneficial for oxide-ion conduction [20,21]. A specific notation is introduced to highlight the presence of oxygen vacancies. Chemical formula is commonly written as LaBaCo_2_O_5+δ_ instead of LaBaCo_2_O_6-δ_ in order to emphasize this high oxygen content deficiency and it does not have to be mixed with the brownmillerite notation [22].

Several authors have reported on the thermal expansion of cation disordered La_0.5_Ba_0.5_CoO_3-δ_ material [7,23], but no data are available for A-site cation ordered LaBaCo_2_O_5+δ_. Chemical expansion of these materials have not been reported to the best of our knowledge. High thermal and chemical expansions are critical for the thermo-mechanical stability of electrochemical devices such as SOFC and gas separation membranes [24,25,26,27]. Significant mismatch in thermal expansion between membranes and sealing materials or an electrode and an electrolyte in a SOFC will induce stresses during thermal cycling. Moreover, chemical expansion gives additional stresses when the material is exposed to a gradient in the chemical potential of oxygen, which is detrimental for the mechanical stability of high temperature electrochemical devices [28].

Here, we report a systematic study of the thermal evolution of the crystal structure, oxygen non-stoichiometry and electrical properties of LaBaCo_2_O_5+δ_ and La_0.5_Ba_0.5_CoO_3-δ_. Particular attention is given to the effect of both cation and oxygen vacancy ordering. The data are discussed in detail in order to correlate the changes of the crystal structure observed by high temperature X-ray diffraction and electrical conductivity with the oxygen deficiency observed by thermogravimetrical analysis.

## 2. Results

**Figure 1:**
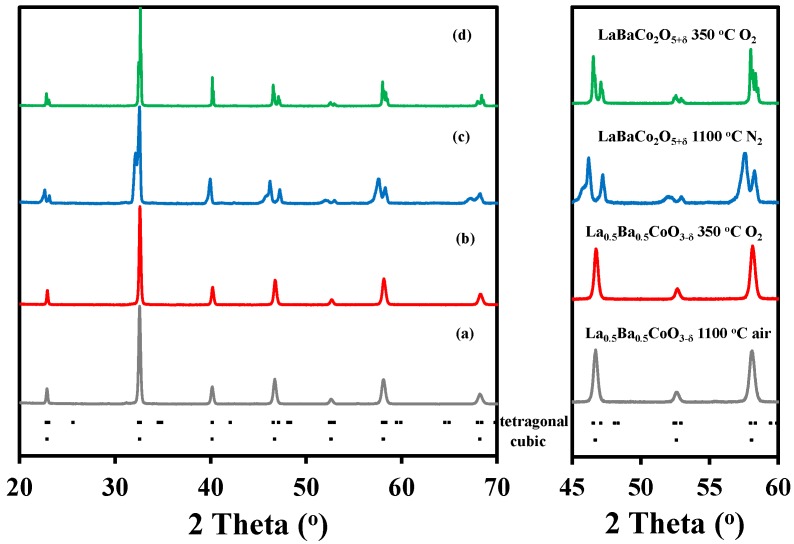
Full range and selected range X-ray diffraction patterns recorded at room temperature of: (**a**) La_0.5_Ba_0.5_CoO_3-δ_ after treatment at 1100 °C for 12 h in air; (**b**) La_0.5_Ba_0.5_CoO_3-δ_ after treatment at 350 °C for 24 h in O_2_; (**c**) LaBaCo_2_O_5+δ_ after treatment at 1100 °C for 12 h in N_2_; and (**d**) LaBaCo_2_O_5+δ_ after treatment at 350 °C for 24 h in O_2_. The Bragg reflections for both tetragonal and cubic cells are included at the bottom.

*Ex-situ* X-ray diffraction (XRD) patterns of La_0.5_Ba_0.5_CoO_3-δ_ and LaBaCo_2_O_5+δ_ after annealing in pure O_2_ are shown in Figure 1. The La_0.5_Ba_0.5_CoO_3-δ_ perovskite synthesized by calcination of the precursor powder obtained by spray pyrolysis and thermally annealed at 1100 °C for 12 h in air was single phase material (Figure 1a) that could be indexed to a simple cubic perovskite with space group $Pm\bar{3}m$. The oxygen stoichiometry of the material after annealing at 350 °C in oxygen was La_0.5_Ba_0.5_CoO_2.98±0.01_, determined by thermogravimetry reduction in 5% H_2_ in N_2_ to its metallic and oxide components, which was confirmed by XRD data. In order to obtain the A-site cation ordered layered double perovskite LaBaCo_2_O_5+δ_, the precursor powder was calcined at 1100 °C for 12 h in N_2_. This calcination step led to a material with high oxygen deficiency and poor crystallinity (Figure 1c). Annealing of this material in pure O_2_ at 350 °C for 24 h resulted in a single phase material with significantly improved crystallinity (Figure 1d), and the diffraction pattern could be indexed in a tetragonal symmetry with space group *P4/mmm*. The absolute oxygen content of this double perovskite annealed in O_2_ was determined by thermal gravimetrical analysis to LaBaCo_2_O_6.00±0.01_. The full reduction of the material was confirmed by XRD data.

Rietveld refinements of the X-ray diffraction data at room temperature confirmed, the cubic structure of the A-site cation disordered La_0.5_Ba_0.5_CoO_2.98_, and a tetragonal structure of the layered double perovskite LaBaCo_2_O_6.00_ showing A-site cation ordering. These findings are in good agreement with the previous works by Rautama *et al.* [29]. Further details about the Rietveld refinement analysis are given below. 

**Figure 2:**
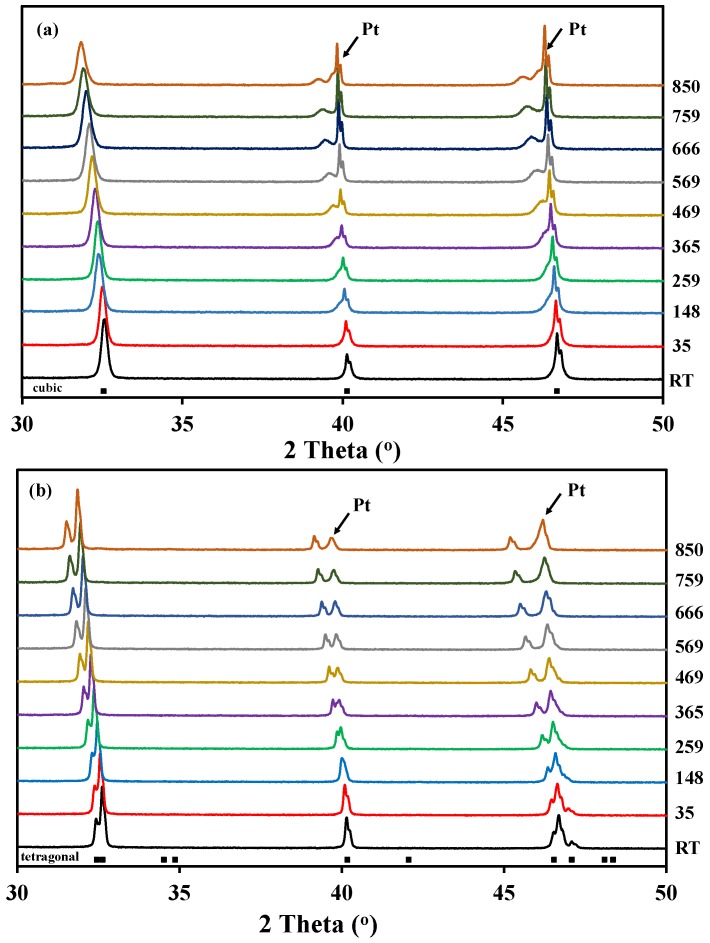
High temperature X-ray diffraction patterns measured in pure O_2_ from room temperature to 850 °C for:(**a**) A-site cation disordered La_0.5_Ba_0.5_CoO_3-δ_; and (**b**) A-site cation order and oxygen vacancy disordered LaBaCo_2_O_5+δ_. The Bragg reflections for both cubic and tetragonal cells are included at the bottom.

Figure 2 shows the high temperature X-ray diffraction data collected for both La_0.5_Ba_0.5_CoO_3-δ_ and LaBaCo_2_O_5+δ_ in O_2_. In the case of La_0.5_Ba_0.5_CoO_3-δ_, the structure remains cubic in the whole temperature range, and both A-site cations and oxygen vacancies formed upon heating remain disordered (Figure 2a). In the case of the layered perovskite, the material remains tetragonal and cation ordered in the whole temperature range while oxygen vacancies formed upon heating were disordered (Figure 2b). The Bragg reflections (Figure 2) are shifted to lower 2θ with increasing temperature due to thermal expansion, but the shift becomes more pronounced at high temperature due to both thermal and chemical expansion. This expansion was observed in all the atmospheres.

**Figure 3:**
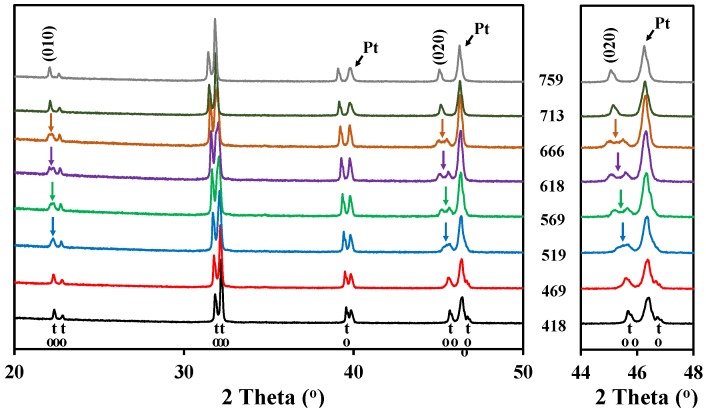
High temperature X-ray diffraction patterns for the layered double perovskite LaBaCo_2_O_5+δ_ in N_2_ from 418 °C to 759 °C. The inset shows the range of temperatures where the splitting of (020) is observed. At the bottom of the figure, the Bragg reflections for both tetragonal and orthorhombic structures of LaBaCo_2_O_5+δ_ are shown.

The high temperature X-ray diffraction data collected for LaBaCo_2_O_5+δ_ in N_2_ are shown in Figure 3. A shift of the Bragg reflection to lower 2θ was also observed due to both thermal and chemical expansion, but in addition two phase transitions were observed during heating in N_2_. A change to a lower symmetry was observed between 450 °C and 675 °C due to the splitting of the (010), (012) and (020) reflections, as marked with an arrow for (010) and (020) in Figure 3. In this range of temperatures, the XRD patterns can be indexed to an *a_p_* × 2 *a_p_* × 2 *a_p_* orthorhombic space group *Pmmm* previously shown by Rautama *et al.* [15] for LaBaCo_2_O_5.5_, which takes into account the ordering of the oxygen vacancies in addition to A-site cation ordering.

**Figure 4:**
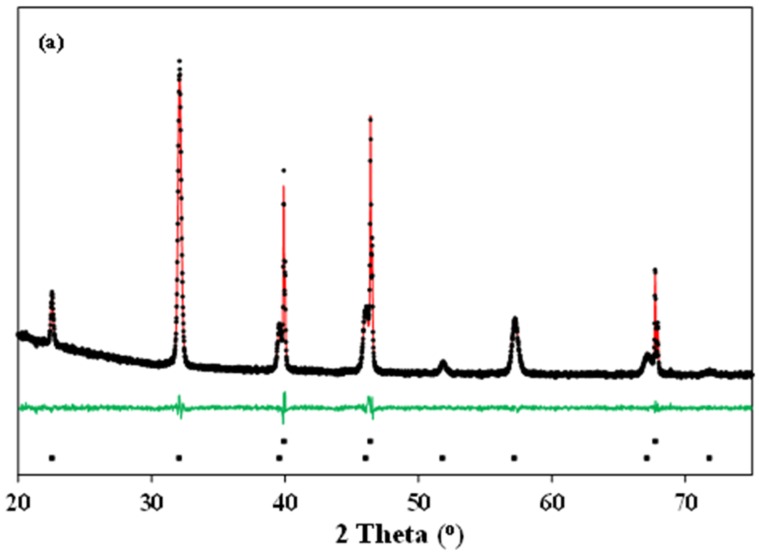

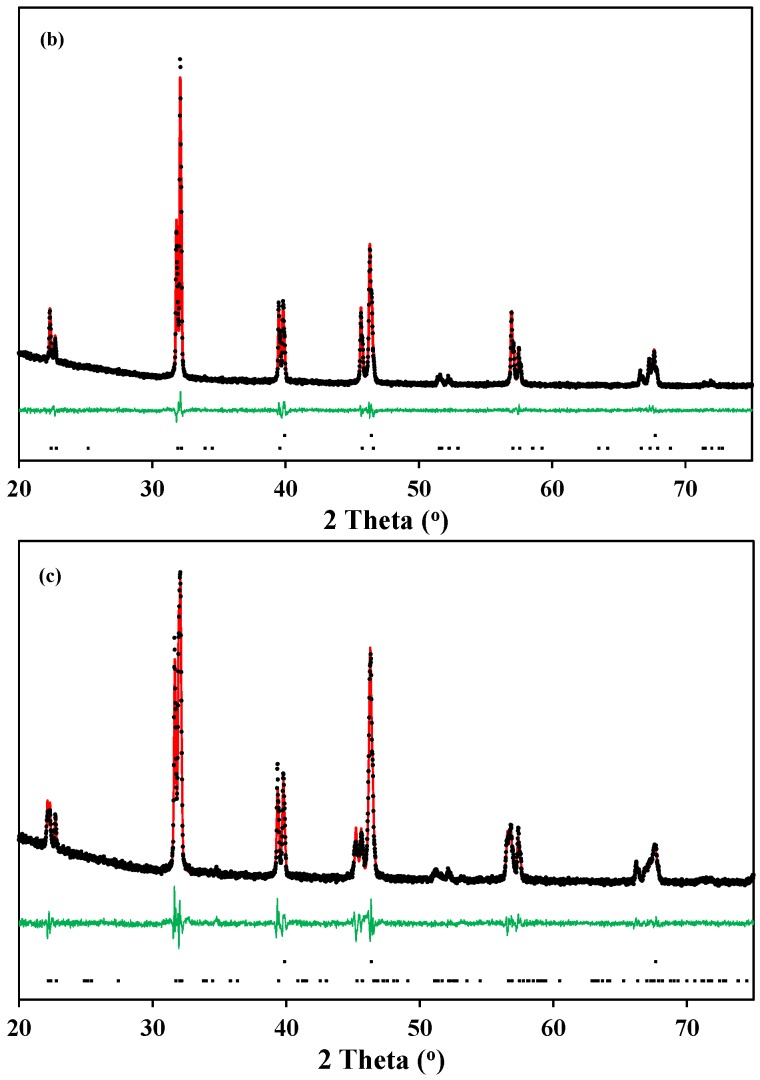
Typical Rietveld fit of the X-ray diffraction patterns of: (**a**) cubic ($Pm\bar{3}m$) La_0.5_Ba_0.5_CoO_3-δ_ at 600 °C in O_2_; (**b**) tetragonal (*P4/mmm*) LaBaCo_2_O_5+δ_ at 600 °C in O_2_; and (**c**) orthorhombic (*Pmmm*) LaBaCo_2_O_5+δ_ at 600 °C in N_2_. The Bragg reflections of Pt and either La_0.5_Ba_0.5_CoO_3-δ_ or LaBaCo_2_O_5+δ_ are included at the bottom of each figure.

Typical Rietveld refinements of the X-ray diffraction patterns of La_0.5_Ba_0.5_CoO_3-δ_ and LaBaCo_2_O_6-δ_ materials at 600 °C in O_2_ and N_2_ are shown in Figure 4. The Rietveld refinement analysis confirms the cubic (Figure 4a), tetragonal (Figure 4b) and orthorhombic (Figure 4c) crystal structures at both room and high temperature, as suggested above. The crystallographic data determined by the refinements are summarized in Table 1.

**Figure 5:**
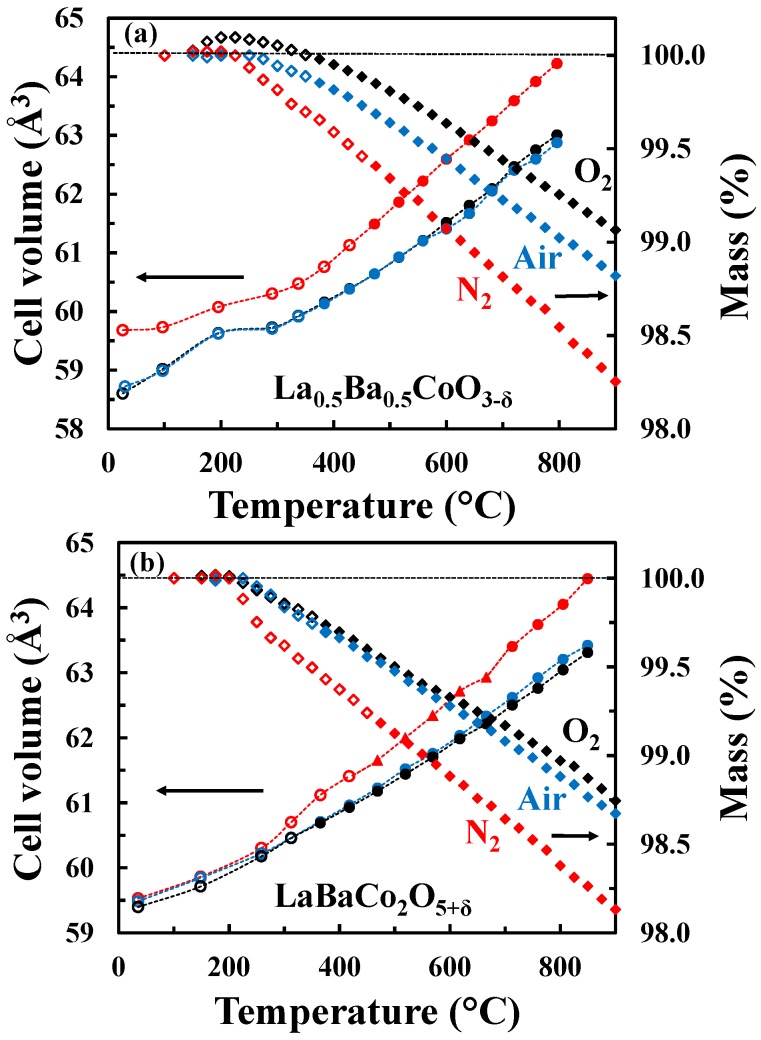
Unit cell volume (circles for tetragonal polymorph and triangles for orthorhombic polymorph) and relative mass change (diamonds) of (**a**) La_0.5_Ba_0.5_CoO_3-δ_ and (**b**) LaBaCo_2_O_5+δ_ as a function of temperature in O_2_ (black symbols), air (blue symbols) and N_2_ (red symbols). Open symbols represent data out of thermodynamic equilibrium. The dotted lines are guides to the eye.

The unit cell volume as well as the mass change as a function of temperature for both the A-site disordered (Figure 5a) and ordered (Figure 5b) materials in O_2_, air and N_2_ are shown in Figure 5. In the case of the A-site disordered material in O_2_, a small mass gain is observed at low temperatures due to oxidation reflecting a small initial oxygen deficiency. This oxidation is not observed in the case of LaBaCo_2_O_5+δ_ because the material was fully oxidized. It is also important to notice that the A-site ordered material demonstrates a larger mass loss than the A-site disordered counterpart. The onset of the thermal reduction is observed as low as ~250 °C, which reflects the high mobility of oxygen anions in both materials and creation of oxygen vacancies. Finally, the onset of the thermal reduction is at a slightly lower temperature for the A-site ordered material than for the A-site disordered one.

**Figure 6:**
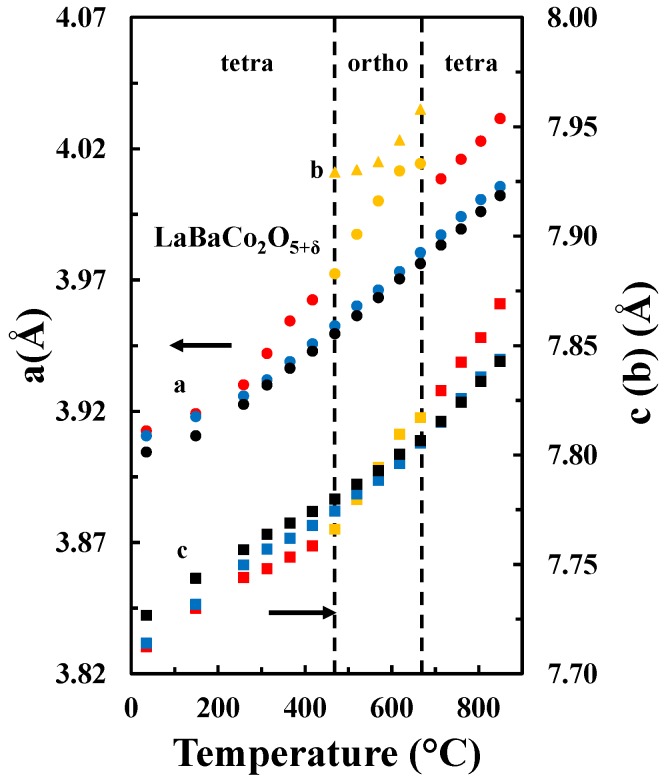
*a* (circles) and *c* (squares) tetragonal cell parameters and *a* (orange circles), *b* (orange triangles) and *c* (orange squares) orthorhombic cell parameters as a function of temperature in O_2_ (black), air (blue) and N_2_ (red and orange) for LaBaCo_2_O_5+δ_.

The temperature dependence of the unit cell parameters for the A-site ordered material in the three different atmospheres is included in Figure 6. For all atmospheres, the unit cell parameter *a* expands more rapidly relative to the *c* parameter, which demonstrates the strong anisotropic chemical expansion of the material. In addition, the cell parameters a and c show different behavior in the different atmospheres. The *a* parameter is smaller for higher pO_2_, independently on the temperature. However, the *c* parameter is smaller at lower temperatures and pO_2_, while at higher temperatures the opposite behavior is apparent.

**Figure 7:**
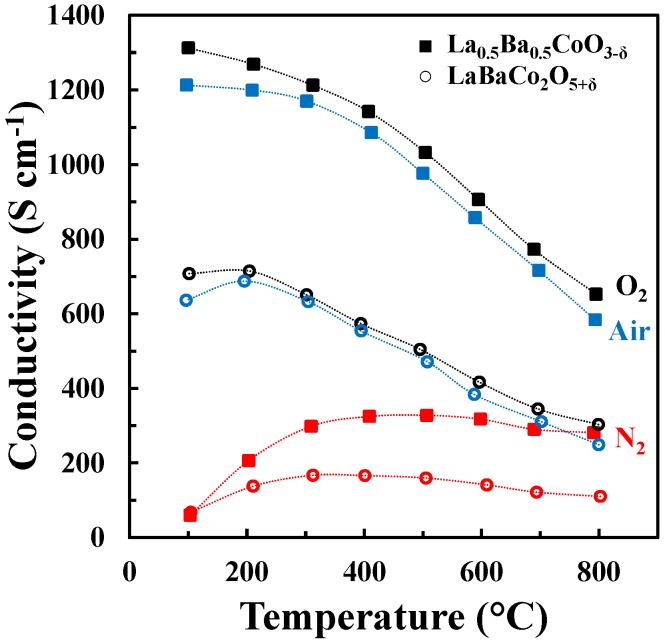
Four-point DC conductivity as a function of temperature for La_0.5_Ba_0.5_CoO_3-δ_ (closed squares) and LaBaCo_2_O_5+δ_ (open circles) in O_2_ (black), air (blue) and N_2_ (red). The dotted lines are guides to the eye.

The DC conductivity of La_0.5_Ba_0.5_CoO_3-δ_ and LaBaCo_2_O_5+δ_ as a function of temperature in N_2_, air and O_2_ is shown in Figure 7. Both materials have a high electronic conductivity (~1000 and 500 S cm^−1^ at 500 °C for La_0.5_Ba_0.5_CoO_3-δ_ and LaBaCo_2_O_5+δ_, respectively) in air and O_2_. The conductivity decreases with increasing temperature, pointing to a metallic behavior, at least at low temperature where thermal reduction will not contribute. The conductivities remain high (~300 and 175 S cm^−1^ at 500 °C for La_0.5_Ba_0.5_CoO_3-δ_ and LaBaCo_2_O_5+δ_, respectively) in N_2_. In this case, the conductivity increases with temperature at lower temperatures (100 °C–400 °C) and is nearly constant at higher temperatures. In addition, the layered perovskite shows nearly half of the conductivity relative to the cubic perovskite, which demonstrate that the A-site cation ordering is not beneficial for the overall electronic conductivity.

## 3. Discussion

### 3.1. Crystal Structure: Ordering Effects

Despite the high potential of La_0.5_Ba_0.5_CoO_3+δ_ and LaBaCo_2_O_5+δ_ to be used as SOFC cathodes [30,31], detailed studies of the thermal evolution of crystal structure and point defects at the SOFC operational temperatures have not appeared in literature to the best of our knowledge. This is probably due to the difficulties in synthesizing single phase LaBaCo_2_O_5+δ_. Kim *et al.* [32] studied high temperature crystal structures of equivalent *Ln*BaCo_2_O_5+δ_ materials with *Ln* = La, Pr, Nd and Sm where *Ln* = La was not reported as A-site cation ordered material but disordered with a cubic $Pm\bar{3}m$ structure.

**Figure 8:**
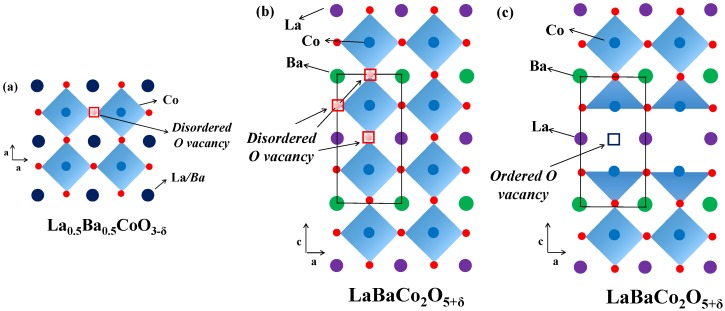
Crystal structure representation of: (**a**) cubic $Pm\bar{3}m$ single perovskite La_0.5_Ba_0.5_CoO_3-δ_; (**b**) tetragonal *P4/mmm* A-site cation ordered and oxygen vacancy disordered LaBaCo_2_O_5-δ_; and (**c**) orthorhombic *Pmmm* both A-site cation and oxygen vacancy ordered LaBaCo_2_O_5-δ_. Dark blue circles are indistinguishable La/Ba atoms, violet balls are La atoms, green balls are Ba atoms, light blue balls are Co atoms, red balls are oxygen, disordered oxygen vacancies are red squares and ordered oxygen vacancies are violet squares.

Here we confirmed that the layered double perovskite LaBaCo_2_O_5+δ_ can be synthesized by thermal treatment of the single perovskite La_0.5_Ba_0.5_CoO_3-δ_ at the appropriate temperature and pO_2_ conditions. Figure 8 shows the different crystal structures of the single perovskite La_0.5_Ba_0.5_CoO_3-δ_ and the layered double perovskite LaBaCo_2_O_5+δ_ adopt as a function of the A-site cation and/or anion ordering schemes. King *et al.* [11] reported that one driving force for A-site cation ordering in *Ln*_0.5_Ba_0.5_*M*O_3-δ_ is the reduction of *M*, the loss of oxygen from the lattice and the creation of oxygen vacancies. The point defect equilibrium that describes the formation of oxygen vacancies, using Kröger–Vink notation [33], is:

(1) $$2Co_{Co}^{.}+O_{O}^{x}\to 2Co_{Co}^{x}+V_{O}^{..}+\frac{1}{2}O_{2}\left(g\right)$$

where the double positively effective charged oxygen vacancies are denoted as $V_{O}^{..}$, positively effective charged $Co_{Co}^{.}$ is Co^4+^ at the cobalt site and neutrally effective charged $Co_{Co}^{x}$ is Co^3+^ at the cobalt site.

The oxygen content is critical to promote A-site cation ordering in LaBaCo_2_O_5+δ_ [15,34,35], but the necessary level of oxygen vacancies that is needed in order to promote A-site cation ordering is not clear from previous studies. Here, we obtained the A-site cation ordered LaBaCo_2_O_5+δ_ from the disordered La_0.5_Ba_0.5_CoO_3-δ_. The critical oxygen vacancy concentration at which the layered double perovskite becomes thermodynamically favorable can be estimated by the TGA data of La_0.5_Ba_0.5_CoO_3-δ_ in N_2_ (conditions at which LaBaCo_2_O_5+δ_ was synthesized). This oxygen content is ~2.64 at 1100 °C, which leads to an average oxidation state for Co of +2.8 (20% Co^2+^ + 80% Co^3+^). Therefore, the ability of the material to adopt such a high level of oxygen vacancies can be the origin of the A-site cation ordering.

**Figure 9:**
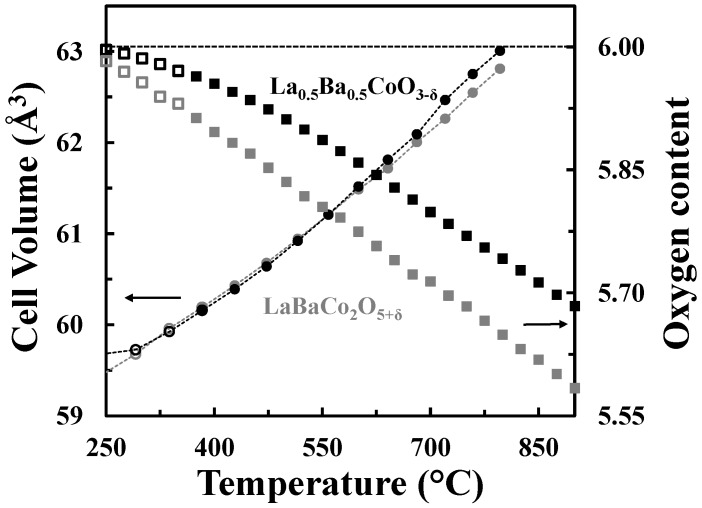
Cell volumes (circles) and oxygen contents (squares) *versus* temperature for La_0.5_Ba_0.5_CoO_3-δ_ (black) and LaBaCo_2_O_5+δ_ (grey) in O_2_. The oxygen content is with respect to the LDP. The dotted lines are guides to the eye.

The A-site cation ordered structure can accommodate oxygen vacancies without extensive chemical expansion as shown in Figure 9, where the cell volumes and the oxygen contents *vs.* temperature in O_2_ for both La_0.5_Ba_0.5_CoO_3-δ_ and LaBaCo_2_O_5+δ_ materials are represented. The unit cell volumes of the two materials are quite similar despite the difference in oxygen deficiency.

**Figure 10:**
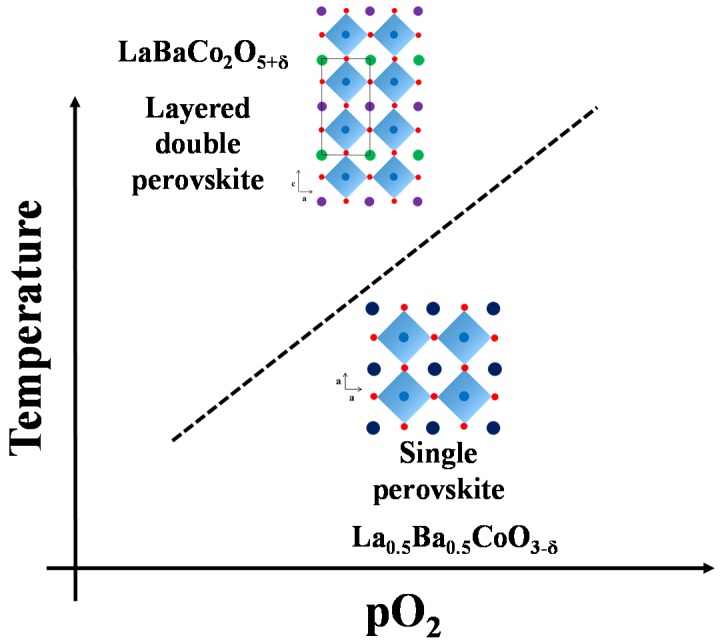
Schematic stability diagram for La_0.5_Ba_0.5_CoO_3-δ_ and LaBaCo_2_O_5-δ_ as a function of temperature and pO_2_.

A schematic phase stability diagram for the phase transition from the disordered La_0.5_Ba_0.5_CoO_3-δ_ to the ordered LaBaCo_2_O_5+δ_ is illustrated in Figure 10. The cation ordering is stabilized at high temperature and low partial pressure of oxygen relative to the cation disordered phase. At high level of oxygen deficiency, the material prefers to adopt a cation ordered layered structure in order to compensate for the oxygen vacancy formation. At lower temperatures and higher oxygen pressure, LaBaCo_2_O_5+δ_ is metastable relative to La_0.5_Ba_0.5_CoO_3-δ_. The average oxidation state of Co and the oxygen vacancy concentration are therefore higher in the metastable state (Figure 9).

**Figure 11:**
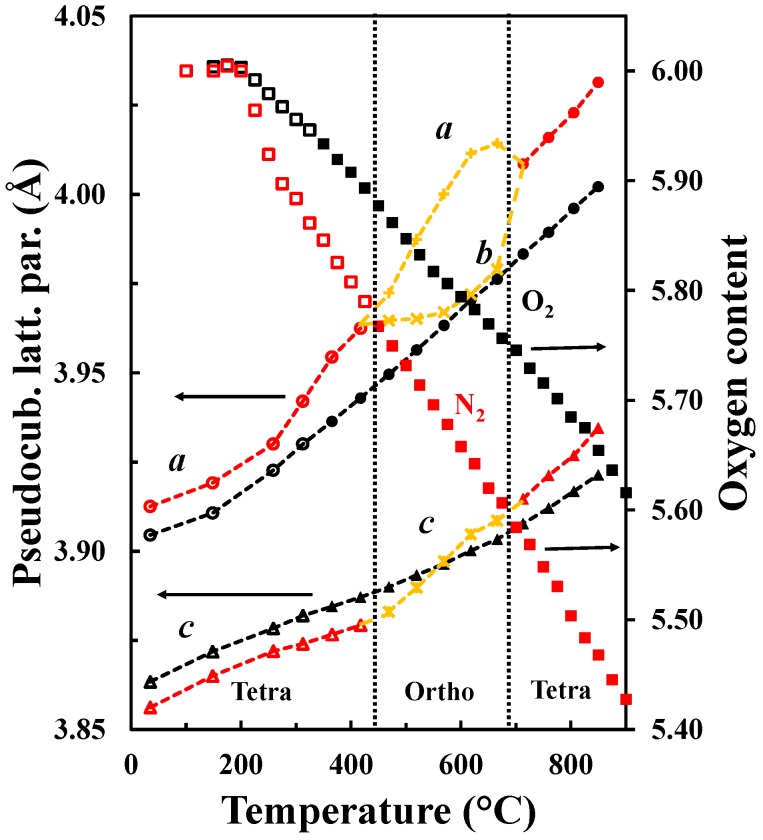
*a* (circles) and *c* (triangles) pseudocubic tetragonal lattice parameters as well as *a* (plusses), *b* (crosses) and *c* (crossed plusses) pseudocubic orthorhombic lattice parameters and oxygen content (squares) *versus* temperature of LaBaCo_2_O_5+δ_ in O_2_ (black) and N_2_ (red). The transitions tetragonal-orthorhombic-tetragonal are shown. Open symbols are data out of the equilibrium. The hatched lines are guides to the eye.

The oxygen vacancy content determines the crystal structure of *Ln*Ba*M*_2_O_5+δ_. The orthorhombic symmetry is generally induced in *Ln*BaCo_2_O_5+δ_ with an oxygen content about 5.5 and temperatures below 500 °C [13]. Tsvetkov *et al.* [36] reported a similar behavior for PrBaCo_2_O_5+δ_ where a transition from *P4/mmm* to *Pmmm* was observed at low pO_2_, 500 °C and 5.5 in oxygen content. For oxygen contents below 5.5, a larger tetragonal cell with a 3a_p_ × 3a_p_ × 2a_p_ supercell is adopted [37]. Transition from a tetragonal structure to an orthorhombic structure with a later transition to the original tetragonal structure together with the oxygen content variation is shown in Figure 11. The first transition from *P4/mmm* to *Pmmm* space groups is proposed to be due to the ordering of the oxygen vacancies upon heating (under conditions where the vacancy concentration is increasing), while the transition from *Pmmm* to *P4/mmm* corresponds to the order-disorder transition of the oxygen vacancies as reported by Kim *et al.* for similar materials [32]. The oxygen vacancy ordering depends both on the oxygen contents and temperatures and is typically observed for oxygen content between 5.65 and 5.70 and in the temperature range 450 °C–650 °C. Although it has been proposed in *Ln*Ba*M*_2_O_5+δ_ that A-site cation ordering is promoted by oxygen vacancy formation [11] and that the substitution of Ba by Sr reduces the tendency to form ordered materials [38,39,40], the driving force for oxygen vacancy ordering is still a matter of discussion. We propose that a stronger tendency for oxygen vacancy ordering will be promoted as the difference in size between *Ln* and Ba becomes larger in order to more easily compensate for the difference of size between them. The ordering results in location of the oxygen vacancies in the *Ln*O layer [15,29]. Furthermore, the level of oxygen deficiency is strongly dependent on the size and type of lanthanide at the A-site and, as La is the largest lanthanide element, we propose that the oxygen deficiency does not have to be as large as 5.5 as in PrBaCo_2_O_5+δ_ [36] in order to compensate for the size difference.

### 3.2. Thermal/Chemical Expansion and Electrical Conductivity

High thermal as well as chemical expansions are among the main challenges in order to utilize La_0.5_Ba_0.5_CoO_3+δ_ and LaBaCo_2_O_5+δ_ as SOFC cathodes. Thermal expansion coefficients (TEC) calculated from linear fit of the cell parameters in O_2_ and N_2_ are summarized in Table 2 for both materials. The A-site cation ordering does not have significant impact on the TEC values. TEC of LaBaCo_2_O_5+δ_ is higher than for other LnBaCo_2_O_5+δ_ materials such as NdBaCo_2_O_5+δ_ [2] and GdBaCo_2_O_5+δ_ [41]. The values reported for La_0.5_Ba_0.5_CoO_3-δ_ are in good agreement with previous reports [7].

As it was discussed in the previous section, the pseudocubic lattice parameters and the oxygen content of LaBaCo_2_O_5+δ_ as a function of temperature in both O_2_ and N_2_ are shown in Figure 11. The corresponding change in the oxygen deficiency is also included in the figure. An anisotropic chemical expansion evolves at the onset of thermal reduction. The thermal reduction occurs at temperatures as low as 250 °C. Chemical expansion coefficient can be estimated from the combination of the unit cell parameters and the oxygen non-stoichiometry. The effect of chemical expansion of the lattice parameter a can be expressed through the chemical strain $\varepsilon_{c}=\frac{\left(a-a_{o}\right)}{a_{o}}=\frac{∆a}{a_{o}}$ where *a*_o_ is the lattice parameter in pure O_2_ and *a* is the corresponding value in for example N_2_. Correspondingly the *normalized chemical strain* [42] can be defined as $\frac{\varepsilon_{c}}{∆\delta }$ where Δδ is the difference in oxygen non-stoichiometry by the change in atmosphere at constant temperature. The chemical strain calculated from the data in Figure 11 are summarized in Table 3. The chemical strain is relatively independent on temperature for both La_0.5_Ba_0.5_CoO_3-δ_ and LaBaCo_2_O_5+δ_. The chemical expansion of La_0.5_Ba_0.5_CoO_3-δ_ is larger than for the A-site cation ordered LaBaCo_2_O_5+δ_ as it can be seen for the cell volume values in Table 3. In addition, the chemical expansion of the A-site cation ordered LaBaCo_2_O_5+δ_ is anisotropic. The chemical expansion of LaBaCo_2_O_5+δ_ in the *a* direction is larger than in the *c* direction but comparable with the chemical expansion of La_0.5_Ba_0.5_CoO_3-δ_ in the *a* direction. Therefore, this anisotropy could be at the origin of the reduction of the chemical expansion for the A-site cation ordered LaBaCo_2_O_5+δ_ material.

Tsvetkov *et al.* [36] also reported an anisotropic chemical expansion at high temperatures for PrBaCo_2_O_5+δ_. However, in this case both *a* and *c* parameters balance out the total chemical expansion leading to a linear increase of the cell volume with no contribution of the chemical expansion to the total expansion. However, for LaBaCo_2_O_5+δ_ evidenced from the cell volume parameter, the expansion of the *a* parameter dominates the global chemical expansion as the cell volume increases with temperature (Figure 5b).

The thermal and chemical expansion coefficients of both La_0.5_Ba_0.5_CoO_3-δ_ and LaBaCo_2_O_5+δ_ can also be compared with other cobaltite materials. LaCoO_3_ has a thermal expansion coefficient slightly lower than the two materials studied in this work [43]. Chen *et al.* [44] reported thermal and chemical expansion coefficients for analogous cobaltites with Sr instead of Ba on the A-site: La_1-x_Sr_x_CoO_3-δ_ with x = 0.3 and 0.4. For x = 0.4, both thermal and chemical expansion coefficients are lower than the values for La_0.5_Ba_0.5_CoO_3-δ_ and LaBaCo_2_O_5+δ_.

At temperatures below ~400 °C, the electrical conductivity of La_0.5_Ba_0.5_CoO_3-δ_ and LaBaCo_2_O_5+δ_ is decreasing with temperature and the materials show a metallic type behavior (Figure 7). Above this temperature region, the electric conductivity of La_0.5_Ba_0.5_CoO_3-δ_ and LaBaCo_2_O_5+δ_ is consistent with previous reports on p-type semiconductor behavior [32,33,34]. Assuming LaCoO_3_ as the host material, substitution of Ba into LaCoO_3_ can be described by defect equilibrium (2), using Kröger–Vink notation [33]:

(2) $$BaCoO_{3}\;\overset{LaCoO_{3}}{\to }\;Ba_{La}^{\prime }+Co_{Co}^{.}+3\;O_{O}^{x}$$

where the negatively effective charged $Ba_{La}^{\prime }$ represents Ba^2+^ on La^3+^ sites and the positively effective charged $Co_{Co}^{.}\;$represents the change of oxidation state of Co from Co^3+^ in LaCoO_3_ to Co^4+^ in BaCoO_3_. $Co_{Co}^{.}$ is inferred as the dominating charge carrier explaining the p-type semiconducting behavior. The concentration of $Co_{Co}^{.}$ is strongly affected by the thermal reduction of Co, as described by defect equilibrium (1).

**Figure 12:**
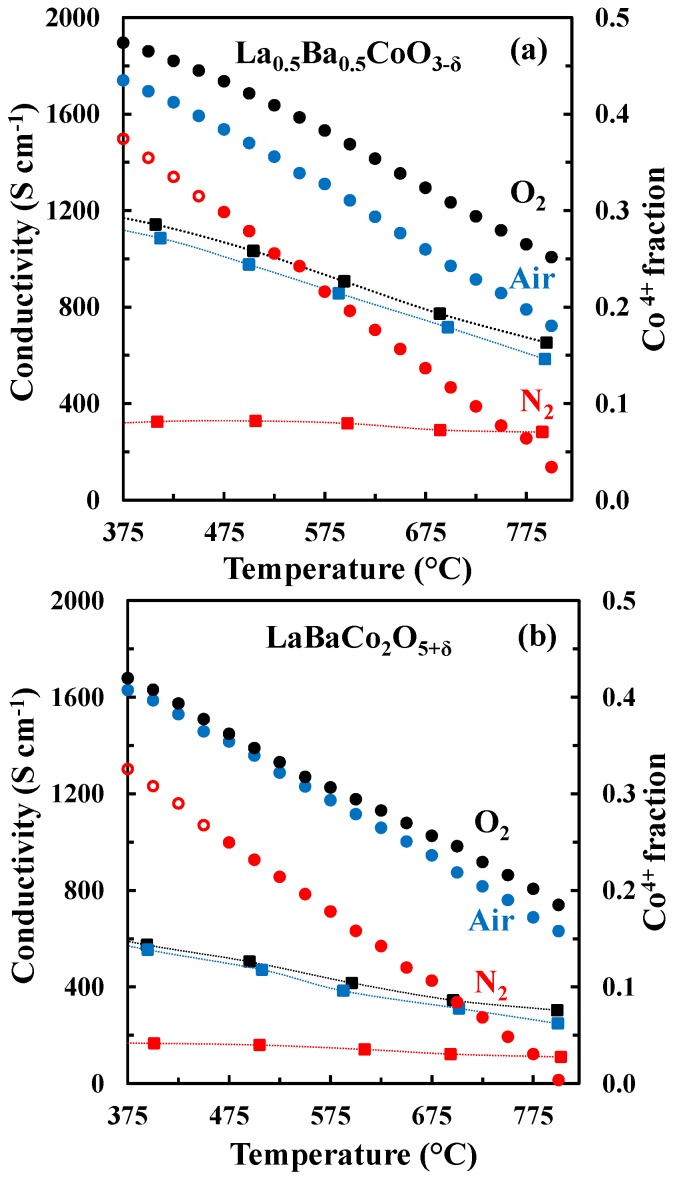
Electrical conductivity (squares) and Co^4+^ fraction (circles) *vs.* temperature for La_0.5_Ba_0.5_CoO_3-δ_ (**a**) and LaBaCo_2_O_5+δ_ (**b**) in O_2_, air and N_2_. The open symbols are data out of the equilibrium. The dotted lines are guides to the eye.

The change in the electronic conductivity is caused by the change in carrier concentration and the increased charge carrier mobility with increasing temperature. The fraction of Co^4+^ and the concentration of active charge carriers $Co_{Co}^{.}$, for La_0.5_Ba_0.5_CoO_3-δ_ and LaBaCo_2_O_5+δ_ calculated from the oxygen non-stoichiometry are plotted in Figure 12 together with the electrical conductivity. The fraction of Co^4+^ is strongly reduced with increasing temperature and the reduction in the electrical conductivity in O_2_ and air at elevated temperatures where thermal reduction occur can be rationalized by the fast reduction of charge carriers with increasing temperature. However, in N_2_, the electrical conductivity is almost constant and independent of temperature even though the change in the concentration of the major charge carrier ($Co_{Co}^{.}$) is more abrupt here relative to the more oxidizing conditions. The electrical conductivity of La_0.5_Ba_0.5_CoO_3-δ_ and LaBaCo_2_O_5+δ_ in N_2_ cannot be qualitative explained by only considering the concentration in the major change carrier.

**Figure 13:**
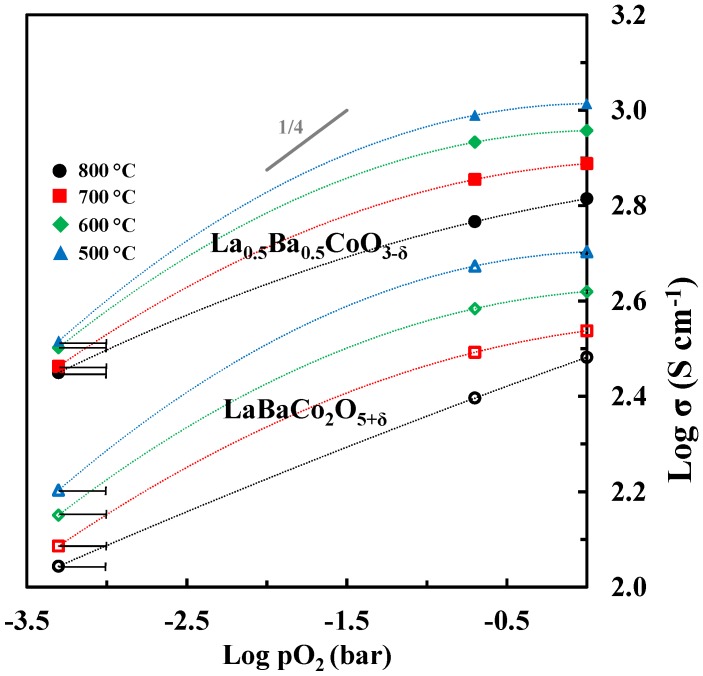
Log σ *vs.* log pO_2_ for La_0.5_Ba_0.5_CoO_3-δ_ and LaBaCo_2_O_5+δ_. At low pO_2_ (N_2_) the uncertainty in pO_2_ is illustrated. The dotted lines are guides to the eye.

The logarithm of electrical conductivity is plotted as a function of the logarithmic partial pressure of oxygen in Figure 13. The electrical conductivity is decreasing with reducing partial pressure of oxygen in line with p-type conductivity inferred from the two proposed defect equilibria (1) and (2). However, the slope of log σ is significantly different from ¼ expected for the region where $Ba_{La}^{\prime }$ is mainly charge compensated by $Co_{Co}^{.}$ and $V_{O}^{..}$ and neglecting the contribution from ionic conductivity. Moreover, in N_2_ the fraction of Co^4+^ approaches zero and one should expect the conductivity to become quite low. Based on the electrical conductivity of acceptor-doped LaCoO_3_ [44] it is not very likely that a classical mass action type model including defect equilibria (1) and (2) and localized electrons will describe accurately the electrical conductivity of Ba doped LaCoO_3_. The electrical conductivity reported here resemble the data reported for other acceptor doped LaCoO_3_ materials where a gradual change from a metallic like conductivity with iterant electrons towards a polaron hopping type mechanism has been discussed [45].

The detrimental effect of the A-site cation ordering on the total electrical conductivity is also evident from the data shown in Figure 13. The lower conductivity by cation ordering can be explained by the anisotropic nature of the crystal structure accompanied by the cation ordering.

## 4. Materials and Methods

### 4.1. Synthesis of the Materials

La_0.5_Ba_0.5_CoO_3-δ_ was prepared by spray pyrolysis (Cerpotech AS, Norway, purity >99%) of mixture of nitrate solutions containing stoichiometric amounts of the cations. The as-received powder was sequentially calcined at 1100 °C for 12 h in air in order to obtain a single phase. A-site cation ordered LaBaCo_2_O_5+δ_ was obtained by calcination of the as-prepared powder by spray pyrolysis at 1100 °C for 12 h in N_2_. In order to control the oxygen content, both La_0.5_Ba_0.5_CoO_3-δ_ and LaBaCo_2_O_5+δ_ materials were finally annealed at 350 °C for 24 h in pure O_2_.

The calcined powders of both La_0.5_Ba_0.5_CoO_3-δ_ and LaBaCo_2_O_5+δ_ were ball milled with 5 mm yttria-stabilized zirconia (YSZ) balls in isopropanol for 72 h in order to remove possible hard agglomerates and sequentially sieved at 250 µm. Rectangular dense bars of typical dimensions 1 × 5 × 20 mm were obtained by uniaxially pressing (20 MPa) of the sieved powders, followed by heat treatment at 1100 °C in air for 4 h for La_0.5_Ba_0.5_CoO_3-δ_ and at 1175 °C in N2 for 4 h for LaBaCo_2_O_5+δ_, achieving 90% of theoretical density.

The particle size of the powders after heat treatment was investigated by scanning electron microscopy (SEM) using a Hitatchi S-3400N instrument.

### 4.2. Structural Characterization at Room and High Temperature

Phase purity for both materials was determined using a Bruker D8 Advance DaVinci X-ray diffractometer. High temperature X-Ray diffraction (HT-XRD) measurements were performed using a Bruker D8 Advance diffractometer equipped with an MRI TCP20 high temperature camera. A Pt strip type resistive heater functioned as sample support. A temperature interval of 100 °C–900 °C and step size of 50 °C were used, with data collected from RT to about 800 °C under different gas atmospheres: air (pO_2_ = 0.2 bar), O_2_ (pO_2_ = 1 bar) and N_2_ (pO_2_ ≈ 10^−4^ bar). An S-type thermocouple was used for temperature determination using the radiant heater. XRD patterns were collected across an angular range 20 °C–75 °C. Total collection time per scan at each temperature was ~30 min. The heating rate between each temperature was 0.1 °C/s. After the set-temperature was reached, a dwell time of 10 min was used before the XRD measurement was started. The sample temperature was calibrated against separate HT-XRD of an alpha-Al_2_O_3_ standard. Rietveld refinements were carried out with the Bruker TOPAS software [46]. For La_0.5_Ba_0.5_CoO_3-δ_ the structure at all temperatures and atmospheres was described using a cubic model ($Pm\bar{3}m$) [29]. For LaBaCo_2_O_5+δ_ the structure in the 450 °C–675 °C temperature range and in N_2_ was described using an orthorhombic model (*Pmmm*) [15] while for the rest of temperatures in N_2_ and all the temperatures in both air and O_2_ the structure was tetragonal (*P4/mmm*) [29]. The peak shape for each pattern was described using a modified Thomson–Cox–Hastings–pseudo-Voigt (PV-TCHZ). For each pattern independent variables consisted of five different parameters: Chebychev polynomial background function, lattice parameters, sample displacement, symmetry constrained atomic positions and isotropic thermal displacement parameters. The oxygen occupancy was fixed to stoichiometric for all the patterns.

### 4.3. Thermogravimetrical Analysis

The absolute oxygen content of La_0.5_Ba_0.5_CoO_3-δ_ and LaBaCo_2_O_5+δ_ was measured by means of thermogravimetric reduction in 5% H_2_ in N_2_ at 1000 °C for 24 h to metallic Co and binary oxides La_2_O_3_ and BaO. The completion of the reduction into these components was confirmed by X-Ray diffraction. The relative oxygen stoichiometry was measured by thermogravimetric measurements using a Netzsch Thermal analysis system 4 (STA449). Measurements were conducted using an Al_2_O_3_ crucible at a heating rate of 10 °C/min. First, the samples were heated to 250 °C and cooled to in order to eliminate any CO_2_ or humidity that can interfere with the measurement. Then, the samples were sequentially heated from 100 °C to 950 °C after every 25 °C, with a dwell time of 35 min in order to reach equilibrium. The time-temperature program corresponded to the program used for the HT-XRD experiments. Data were collected in different atmospheres: air, O_2_ and N_2_ similar to the high temperature XRD measurements were taken. The mass changes prior to the measurement of the samples were corrected for buoyancy by measurements on an empty Al_2_O_3_ crucible. The weight reported at each dwell temperature is the weight recorded at the end of each dwell period of 35 min. The relative changes in the oxygen non-stoichiometry were calculated based on the TGA data.

### 4.4. Electrical Conductivity

Electrical conductivity for La_0.5_Ba_0.5_CoO_3-δ_ and LaBaCo_2_O_5+δ_ was determined using a four-point DC method on sintered bars, as previously described by Wærnhus *et al.* [47] in the same three different atmospheres that previous TGA and HT-XRD were performed: air, O_2_ and N_2_.

## 5. Conclusions

A-site cation ordered and layered double perovskite LaBaCo_2_O_5+δ_ and cubic cation disordered perovskite La_0.5_Ba_0.5_CoO_3-δ_ were obtained from a powder synthesized by spray pyrolysis. LaBaCo_2_O_5+δ_ was prepared by thermal annealing of La_0.5_Ba_0.5_CoO_3-δ_ in N_2_. Thermogravimetric analysis demonstrated that a critical level of oxygen vacancies is required in La_0.5_Ba_0.5_CoO_3-δ_ in order to stabilize the A-site cation ordered structure. The oxygen content of La_0.5_Ba_0.5_CoO_3-δ_ for obtaining A-site ordering was calculated to be ~2.64 and, therefore, LaBaCo_2_O_5+δ_ is metastable with respect to La_0.5_Ba_0.5_CoO_3-δ_ in oxidizing atmosphere. High temperature X-ray diffraction between room temperature and 800 °C revealed that La_0.5_Ba_0.5_CoO_3-δ_ remained cubic at the studied temperatures in O_2_ as well as in N_2_. No structural changes were observed for tetragonal A-site cation ordered LaBaCo_2_O_5+δ_ in air while a change to orthorhombic structure was observed between 475 °C and 650 °C and at low pO_2_ (N_2_) due to ordering of oxygen vacancies. Combining thermogravimetric analyses and high temperature X-ray data also showed that the ordering of oxygen vacancies depends strongly on the level of oxygen deficiency and temperature. The thermal and chemical expansions of the two materials were determined using the high X-ray temperature diffraction data combined with thermogravimetrical analysis. An anisotropic chemical expansion was shown for the A-site cation ordered LaBaCo_2_O_5+δ_. A high electronic conductivity for both A-site cation ordered and disordered materials in both O_2_ (~1000 and 500 S cm^−1^ at 500 °C in pO_2_ = 1 bar for La_0.5_Ba_0.5_CoO_3-δ_ and LaBaCo_2_O_5+δ_, respectively) and N_2_ (~300 and 175 S cm^−1^ at 500 °C for La_0.5_Ba_0.5_CoO_3-δ_ and LaBaCo_2_O_5+δ_, respectively) was evidenced. At low temperatures, a metallic-like conductivity was observed, while a p-type semiconducting conductivity behavior was evident at elevated temperatures.

## Figures and Tables

**Table 1:** Crystallographic data at 600 °C of cubic La_0.5_Ba_0.5_CoO_3-δ_ and tetragonal LaBaCo_2_O_5+δ_ in O_2_; and orthorhombic LaBaCo_2_O_5+δ_ in N_2_: lattice parameters, atomic positions, occupancies, thermal parameters (B_eq_) and goodness of fit (R_wp_).

Space Group	Lattice Par. (Å)	Atom	x	y	z	Occ	B_eq_	R_wp_	R_exp_	χ^2^
$Pm\bar{3}m$	*a* = 3.9410 (2)	La	0	0	0	0.5	3.8 (1)	3.2	2.72	1.18
		Ba	0	0	0	0.5	3.8 (1)			
		Co	0.5	0.5	0.5	1	2.7 (2)			
		O	0	0.5	0.5	1	4.8 (2)			
*P4/mmm*	*a* = 3.9633 (2)	La	0	0	0	1	3.9 (1)	3.38	2.70	1.25
	*c* = 7.7928 (3)	Ba	0	0	0.5	1	3.9 (1)			
		Co	0.5	0.5	0.253 (6)	1	3.0 (1)			
		O1	0.5	0.5	0	1	6.7 (3)			
		O2	0	0.5	0.234 (8)	1	6.7 (3)			
		O3	0.5	0.5	0.5	1	6.7 (3)			
*Pmmm*	*a* = 4.0001 (2)	La	0.5	0.252 (5)	0	1	3.3 (2)	4.18	3.12	1.61
	*b* = 7.9340 (5)	Ba	0.5	0.251 (4)	0.5	1	3.3 (2)			
	*c* = 7.7942 (5)	Co1	0	0	0.253 (7)	1	2.8 (2)			
		Co2	0	0.5	0.240 (6)	1	2.8 (2)			
		O1	0	0.244 (20)	0.204 (5)	1	3.7 (4)			
		O2	0.5	0	0.22 (3)	1	3.7 (4)			
		O3	0.5	0.5	0.22 (3)	1	3.7 (4)			
		O4	0	0	0.5	1	3.7 (4)			
		O5	0	0.5	0.5	1	3.7 (4)			
		O6	0	0.5	0	1	3.7 (4)			

**Table 2:** Thermal expansion coefficients of the unit cell parameters (α_a_ and α_c_) and the isotropic linear thermal expansion coefficient for La_0.5_Ba_0.5_CoO_3-δ_ and LaBaCo_2_O_5+δ_ in O_2_ and N_2_ atmospheres.

	**O_2_**			
Material	T range (°C)	α_a_ (10^−6^ K^−1^)	α_c_ (10^−6^ K^−1^)	α_i_ (10^−6^ K^−1^)
La_0.5_Ba_0.5_CoO_3-δ_	RT-250	19 ± 1		
	300–800	31.3 ± 0.1		
LaBaCo_2_O_5+δ_	RT-200	13 ± 1	18 ± 1	15 ± 1
	250–750	34.6 ± 0.2	16.1 ± 0.3	29.3 ± 0.3
	800–850	36.4 ± 0.2	25.2 ± 0.3	32.8 ± 0.3
	**N_2_**			
Material	T range (°C)	α_a_ (10^−6^ K^−1^)	α_c_ (10^−6^ K^−1^)	α_i_ (10^−6^ K^−1^)
La_0.5_Ba_0.5_CoO_3-δ_	RT-250	15 ± 1		
	300–800	37.4 ± 0.3		
LaBaCo_2_O_5+δ_	RT-200	20 ± 1	18 ± 1	20 ± 1
	250–400	50.9 ± 0.5	13.6 ± 0.4	38.4 ± 7.1
	450–650	53.0 ± 2.6	33.2 ± 0.8	39.3 ± 8.5
	700–850	41.9 ± 0.5	37.1 ± 0.6	40.3 ± 5.4

**Table 3:** Normalized chemical strain values for La_0.5_Ba_0.5_CoO_3-δ_ and LaBaCo_2_O_5+δ_.

	LaBaCo_2_O_5+δ_			La_0.5_Ba_0.5_CoO_3-δ_
	Chemical Strain			Chemical Strain
T (°C)	(Δa/ao)/Δδ	(Δc/co)/Δδ	⅓ (ΔV/Vo)/Δδ	(Δa/ao)/Δδ
450	0.05 ± 0.01	-0.02 ± 0.01	0.02 ± 0.01	0.07 ± 0.01
550	0.07 ± 0.01	0.01 ± 0.01	0.03 ± 0.01	0.07 ± 0.01
650	0.06 ± 0.01	0.01 ± 0.01	0.03 ± 0.01	0.07 ± 0.01
750	0.04 ± 0.01	0.01 ± 0.01	0.03 ± 0.01	0.05 ± 0.01
850	0.04 ± 0.01	0.02 ± 0.01	0.03 ± 0.01	0.06 ± 0.01

## Abbreviations

The following abbreviations are used in this manuscript:

RT: Room Temperature
LDP: Layered double perovskite
SOFC: Solid oxide fuel cells
XRD: X-Ray Diffraction data
YSZ: Yttria-stabilised zirconia
SEM: Scanning electron microscopy
HT-XRD: High temperature x-ray diffraction
PV-TCHZ: Thomson Cox-Hastings pseudo-Voigt
TGA: Thermogravimetric analysis
TEC: Thermal expansion coefficient
